# Olfactory Receptors as Biomarkers in Human Breast Carcinoma Tissues

**DOI:** 10.3389/fonc.2018.00033

**Published:** 2018-02-15

**Authors:** Lea Weber, Désirée Maßberg, Christian Becker, Janine Altmüller, Burkhard Ubrig, Gabriele Bonatz, Gerhard Wölk, Stathis Philippou, Andrea Tannapfel, Hanns Hatt, Günter Gisselmann

**Affiliations:** ^1^Department of Cell Physiology, Ruhr-University Bochum, Bochum, Germany; ^2^Cologne Center for Genomics, University of Cologne, Cologne, Germany; ^3^Center for Molecular Medicine Cologne, University of Cologne, Cologne, Germany; ^4^Clinic for Urology, Augusta-Kranken-Anstalt Bochum, Bochum, Germany; ^5^Clinic of Gynaecology, Augusta-Kranken-Anstalt Bochum, Bochum, Germany; ^6^Clinic of Gynaecology, Herz-Jesu-Krankenhaus Dernbach, Dernbach, Germany; ^7^Department of Pathology and Cytology, Augusta-Kranken-Anstalt Bochum, Bochum, Germany; ^8^Institute for Pathology, Ruhr-University Bochum, Bochum, Germany

**Keywords:** olfactory receptors, biomarker, next-generation sequencing, OR2B6, breast carcinoma, RNA-Seq, pancreatic carcinoma

## Abstract

Olfactory receptors (ORs) are known to be expressed in a variety of human tissues and act on different physiological processes, such as cell migration, proliferation, or secretion and have been found to function as biomarkers for carcinoma tissues of prostate, lung, and small intestine. In this study, we analyzed the OR expression profiles of several different carcinoma tissues, with a focus on breast cancer. The expression of OR2B6 was detectable in breast carcinoma tissues; here, transcripts of OR2B6 were detected in 73% of all breast carcinoma cell lines and in over 80% of all of the breast carcinoma tissues analyzed. Interestingly, there was no expression of OR2B6 observed in healthy tissues. Immunohistochemical staining of OR2B6 in breast carcinoma tissues revealed a distinct staining pattern of carcinoma cells. Furthermore, we detected a fusion transcript containing part of the coding exon of OR2B6 as a part of a splice variant of the histone HIST1H2BO transcript. In addition, in cancer tissues and cell lines derived from lung, pancreas, and brain, OR expression patterns were compared to that of corresponding healthy tissues. The number of ORs detected in lung carcinoma tissues was significantly reduced in comparison to the surrounding healthy tissues. In pancreatic carcinoma tissues, OR4C6 was considerably more highly expressed in comparison to the respective healthy tissues. We detected OR2B6 as a potential biomarker for breast carcinoma tissues.

## Introduction

Olfactory receptors (ORs) belong to the class of G-protein-coupled receptors, consisting of seven transmembrane domains. This superfamily contains approximately 380 functional OR genes, representing the largest gene family in humans (1, 2). It was discovered by Linda Buck and Richard Axel as a gene family that was expressed in olfactory sensory neurons in the rat olfactory epithelium in 1991 (3). Since 1992, several groups have discovered the functional expression of ORs in several healthy human tissues (4–6). Especially in recent years, many milestones were set in the field of ectopic expression by the discovery of the receptor’s function outside of the nose. Ectopically expressed human ORs are capable of mediating sperm motility and chemotaxis, serotonin release, cytokinesis, wound healing, myocardial function, or melanogenesis (7–12). However, their influence is not only of great importance in healthy tissues but also in several cancer tissues. OR51E2 shows a high, tumor-specific expression in prostate cancer cells in comparison to healthy prostate tissues (13, 14). Furthermore, activating the OR by its ligand β-ionone leads to the inhibition of cell proliferation (13).

Ectopically expressed ORs in cancer tissues play a role in multiple pathophysiological processes. In hepatocarcinoma cells, activation of OR1A2 by citronellal leads to calcium signals and a reduction in cell proliferation (15). In non-small-cell lung cancer, OR2J3 expression increases upon helional application, and furthermore, the activation induces apoptosis and inhibits cell proliferation (16). Melanoma cells express the receptor OR51E2, which is upregulated in these cells in comparison to normal melanocytes, indicating that OR51E2 might play a role in early melanoma development and progression (17). Activation by its ligand ß-ionone leads to the decrease of cell migration and the induction of apoptosis. Similar effects are shown for OR51B4 in colorectal cancer cells as well: here, activation of the OR by the specific ligand Troenan induces apoptosis and reduced cell migration and proliferation. Furthermore, the actin filament formation is highly impaired upon stimulation with Troenan (18). In leukemia cells, OR2AT4 is capable of inducing proapoptotic processes upon activation by the ligand Sandalore (19, 20). Here, a cAMP-dependent pathway is induced, leading to the phosphorylation of ERK1/2 and p38 MAPK. Beside the effect on pathophysiological processes, ORs also function as tumor markers. As mentioned before, OR51E2 and OR51E1 show an increased expression in prostate cancer cells, whereas OR51E1 is postulated to be a marker for lung cell carcinoids and cancer of the small intestine (21–23). Another OR, OR7C1, was shown to be a marker for colon cancer-initiating cells and might be a potent immunotherapy target (24). A study by Ranzani et al. demonstrated the expression of at least 1 OR in 49% of all cell lines and furthermore suggest a functional role of OR2C3 in the development or progression of human melanomas (25).

Due to innovative techniques, such as next-generation sequencing (NGS), the knowledge of the medical research potential benefits of ectopically expressed ORs is constantly rising. Several studies describe the broad expression profile of ORs in different human tissues, whereas only a few studies analyze the expression of ORs in cancerous tissues (4, 26). Our study sheds light on the expression patterns of different ORs in tumor tissues; furthermore, it highlights an innovative potential tumor marker for breast cancer.

## Materials and Methods

### Tissue Preparation

7 human breast cancer tissues were obtained from Prof. Dr. Tannapfel, Institute for Pathology, Ruhr-University Bochum. The study was carried out in accordance with the Declaration of Helsinki. A written informed consent was signed by each patient, who participated. The experiments were approved by the Regional Research Ethics Committee (Ethical Committee of the Medical Faculty, Ruhr-University Bochum); Ethic no: 17-6113.

Details on breast carcinoma tissues:

**Table:** 

No.	Tissue type	Age	Location	Grading	Tumor spread (lymph)	Tumor spread (blood vessel)
15	Breast carcinoma	88	Left	pT3/G3/pN2a (9/12)	L1	V0
16	Breast carcinoma	80	Left	pT1c/G2/pNO (0/1 sn)	L0	V0
17	Breast carcinoma	59	Right	pT3/G3/pN1a (2/25; 1/3 sn)	L0	V0
18	Breast carcinoma	58	Right	pT2/G2 pN0 (0/3; 0/1 sn)	L0	V0
19	Ductale carcinoma *in situ*	79	Left	pTis G2	/	/
20	Breast carcinoma	87	Left	pT2/G3/pNO (0/4)	L0	V0
21	Breast carcinoma	82	Left	pT2/G3/pNO (0/2 sn)	L0	V0

Tumor tissues were first preserved in RNAlater (Qiagen, Hilden, Germany) and then disruption was carried out using a Homogenizer (Precellys24, Bertin, Montigny-le-Bretonneux, France) and 1.4 ceramics beads (Precellys^®^). The RNeasy Mini Kit (Qiagen, Hilden, Germany) was used according to the manufacturer’s protocol to isolate the RNA from the tumor samples. In addition, gDNA-Eliminator columns (Qiagen, Hilden, Germany) and an additional on-column DNaseI digestion were used according to the manufacturer’s protocol to avoid genomic contaminations.

### Reverse Transcriptase PCR (RT-PCR)

The expression of the ORs detected *via* RNA-Seq was validated using RT-PCR. RNA was reverse transcribed using the iScript cDNA Synthesis Kit (Bio-Rad Laboratories, Hercules, CA, USA) according to the manufacturer’s instructions. An equivalent of 50 ng of RNA was used for each RT-PCR experiment. The primers detected 200–300 bp of the OR ORF. GoTaq qPCR Master Mix (Promega, Madison, WI, USA) was used according to the manufacturer’s protocol for the PCR reaction. PCR was performed using the Mastercycler realplex^2^ (Eppendorf, Hamburg, Germany) (20 µl total volume, 40 cycles: 95°C, 59°C, 72°C, 45 s each). All experiments were conducted in triplicate.

Primer sequences:

**Table:** 

Primer	Sequence
OR2B6—histone—forward (P1)	AGTGTATATATAAGGGGATTCAGC
OR2B6—histone—reverse (P2)	AGCCTGTGCATTTTGCTTGTAGTC
OR2B6—forward (P3)	GATCGACCTTGGCTGGAGTTT
OR2B6—reverse (P4)	ATATTCAGTAGCCCCCAAG

### Next-Generation Sequencing

Basic NGS analysis was performed as previously described (26). Briefly, for transcriptome analysis, RNA from tissue samples was prepared as stated above. At the Cologne Center for Genomics Next Generation Sequencing unit, libraries for NGS were constructed from mRNA. RNA-Seq was performed on the Illumina HiSeq2000 sequencing platform as paired-end reads with 101-nucleotide length. Raw data were generated for seven breast carcinoma tissue samples in the fastq format. Reads were aligned to the human reference genome hg19 by using Tophat (v1.2.0) (27). Bowtie, a short-read mapping program, was used to arrange the alignments and includes TopHat (28). SAMtools was used to sort and index the output files in the BAM format (29). The calculation of the abundance of the aligned reads was conducted by the program Cufflinks (30). Cufflinks uses the RefSeq reference transcriptome in the Gene Transfer Format obtained from the University of California Santa Cruz Genome Bioinformatics database. The relative abundance of the transcripts for each gene was quantified by Cufflinks using the unit FPKM (fragments per kilobase of exon per million fragments mapped) (27). The visualization of the data sets was conducted using the Integrative Genomic Viewer (http://software.broadinstitute.org/software/igv) to analyze the read distribution.

### Published Raw RNA-Seq Data

Data sets used for the RNA-Seq analysis of various cell lines, tumor tissues and healthy tissues were obtained from the SRA archive. Data sets of brain (31–33), data sets of breast (34–39), data sets of lung: SAMN00194128, data sets of pancreas (40), Accession numbers PRJNA237340 and PRJNA30709.

### Cell Culture

Hana3A cells were used for the recombinant expression of OR2B6 and were kindly provided by H. Matsunami (Duke University Medical Center, Durham, NC, USA). Hana3A cells were cultured in DMEM containing 10% fetal bovine serum, 5 mM Glutamax and 100 units/ml penicillin/streptomycin (Gibco^®^, Life Technologies, Carlsbad, CA, USA). The cells were cultured in a 5% CO_2_ humidified atmosphere at 37°C and were passaged every 3–4 days at a confluence of 90% using TrypLE^TM^ (Life Technologies, Carlsbad, CA, USA).

### Cell Transfection

For the expression of ORs in Hana3A cells, expression vectors were constructed using standard PCR methods. ORs were expressed as fusion proteins containing 20 amino acids of the *N*-terminal region of the bovine rhodopsin (rhodopsin tag) as described elsewhere (41).

To ensure the specificity of the OR antibodies, we used Hana3A cells to express recombinant ORs. Cells were grown on cover slips in 24-well plates and were transfected with Lipofectamine 2000 (Invitrogen, Carlsbad, CA, USA) according to the manufacturer’s protocol. The transfection mixture contained 300 ng of the OR plasmid and 60 ng of the RTP1s plasmid per plate, which enhances the transport of the OR into the cell membrane.

### Immunocytochemistry

The specificity of hOR2B6 antibody (obtained from Abcam, dilution 1:50) was analyzed using immunocytochemistry of OR2B6-transfected Hana3A cells. The OR2B6-transfected cells were grown on coverslips in 24-well cell culture plates and fixed in 4% paraformaldehyde at 4°C for 20 min. Then, cells were washed and permeabilized in PBS^−/−^ + 0.1% Triton X-100, and blocking was conducted in PBST + 1% fish gelatin for 1 h at RT. The purified goat IgG polyclonal antibody against OR2B6 (Abcam, dilution: 1:100 in blocking solution) as well as the monoclonal anti-rhodopsin antibody 4D2 (anti-rho; Abcam; dilution 1:100) were incubated for 2 h at RT. After that, three washing steps were performed with washing solution, and the secondary antibodies coupled to Alexa Fluor 488 (Invitrogen) and 633 were applied for 45 min at RT. Cells were washed three times and covered with Prolong^®^ Gold Antifade reagent (Life Technologies). Images were taken with a Zeiss LSM 510 Meta confocal microscope. The localization of OR2B6 in Hana3A cells was visualized using a purified goat IgG polyclonal antibody against OR2B6 (Santa Cruz, dilution: 1:100 in blocking solution) and mouse monoclonal anti-goat antibody (Abcam, dilution 1:1000 in blocking solution). 4′6-diamidino-2-phenylindole (dilution 1:200) was applied for staining of the cell nuclei.

### Immunohistochemistry

Breast cancer tissue samples were derived from patients undergoing radical mastectomy or punch biopsies. Samples were immediately stored at −80°C for subsequent RNA isolation or immunohistochemistry. Immunohistochemistry of human breast carcinoma tissue paraffin sections was performed on a Ventana BenchMark Ultra instrument (Ventana Medical System, Tucson, AZ, USA). For staining, the UltraView Universal DAB Detection Kit was used according to the manufacturer’s protocol. Primary antibody was diluted 1:50 and incubated for 32 min. Furthermore, all sections were co-stained with hematoxylin to illustrate tissue architecture. Staining was visualized by an Olympus BX 43 microscope.

### Statistics

All results were first tested for normality (Shapiro–Wilk test) and equal variance. If the data had a normal distribution, a two-tailed unpaired Student’s *t*-test was used. Data that were not normally distributed were analyzed using a Mann–Whitney test or a Kruskal–Wallis one-way analysis of variance. The statistical significance is indicated as follows: *p < 0.05, **p < 0.01, and ***p < 0.001.

## Results

### OR Expression Profile in Human Breast Carcinoma Tissues

**Figure 1:**
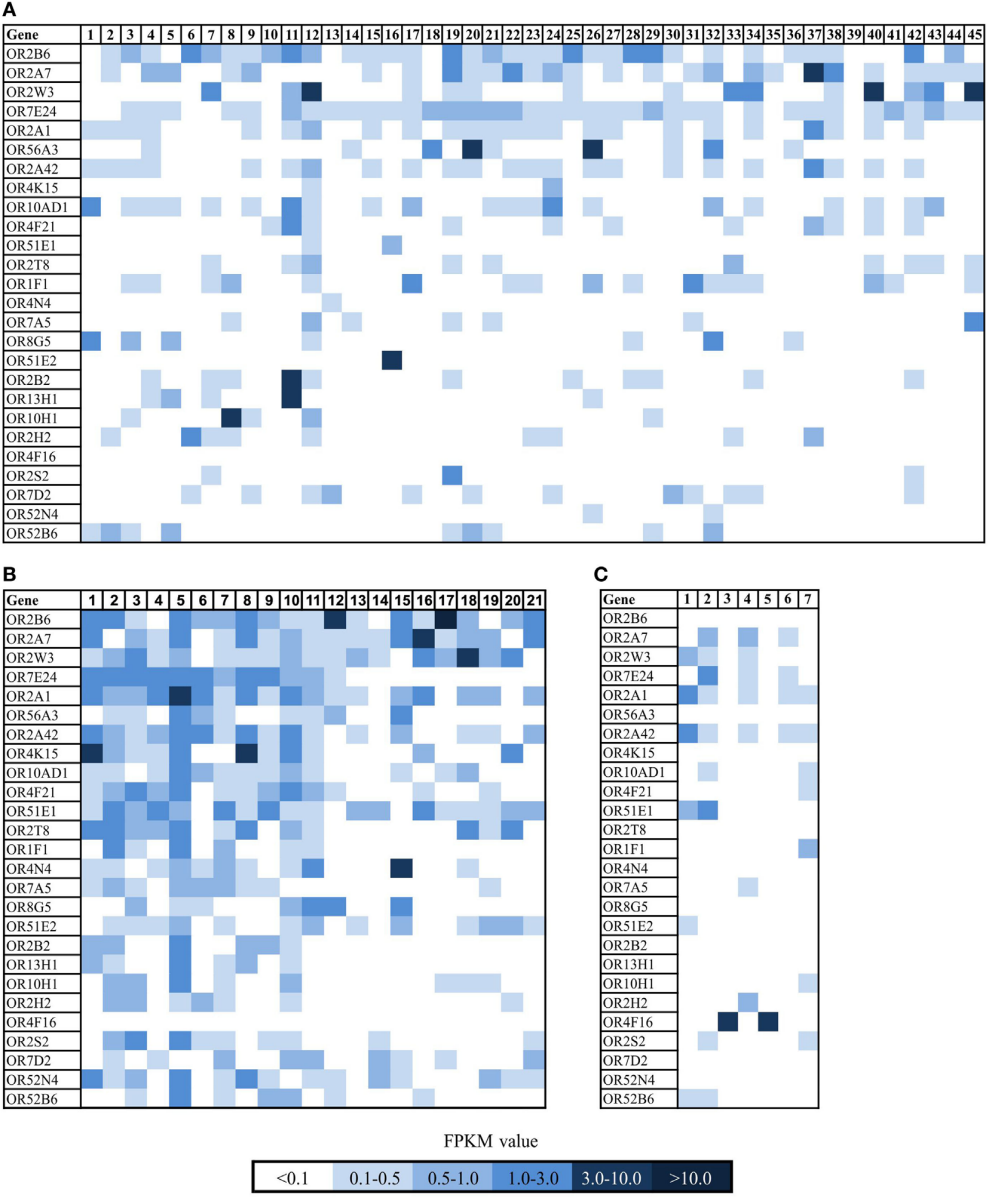
Expression pattern of ectopically expressed olfactory receptors (ORs) in breast carcinoma tissue samples and cell lines and healthy breast tissue samples. **(A)** Heat map shows 26 ORs in 45 breast cancer cell lines. For further details on the cell lines, see Table S1 in Supplementary Material. Sample no. 1 and 45 are self-generated RNA-Seq data of breast cancer cell lines. **(B)** Heat map shows 26 ORs in 21 breast carcinoma tissues. Sample 1–13 are reanalyzed data sets from NCBI (for further details, see Section “Materials and Methods”), samples 15–21 are self-generated RNA-Seq data of breast carcinoma tissue samples. **(C)** Heat map shows 26 ORs in 7 normal breast tissue samples. Color intensity correlates with the expression strength. Dark blue represents FPKM values >3, and light blue represents FPKM values <0.5.

The development of the RNA-Seq technique has revolutionized quantitative expression profiling and contributed substantially to the understanding of ectopically expressed ORs in diverse human tissues (15, 16, 20, 26, 42, 43). This advancing technology led to the accumulation of a large number of transcriptome data sets, which are available for reanalysis for a variety of mRNA-based questioning. We reanalyzed the RNA-Seq data of different human breast carcinoma tissues and breast carcinoma cell lines from the SRA archive to set up an expression profile of ORs in breast carcinoma tissues in comparison to corresponding normal tissues. Furthermore, we collected different breast carcinoma tissue samples and generated RNA-Seq data of seven breast cancer tissues samples. We used the Illumina RNA-Seq protocol to generate 5–10 million reads per transcriptome, with a read length of 101 bp. We analyzed all data using TopHat and Cufflinks software as described in the study by Flegel et al. and mapped the sequence reads to the human reference genome (hg19) (26). Expression intensities of all detected transcripts were calculated using the FPKM value (44). Transcript structures and read distribution were analyzed by visualizing the read alignment using the IGV. We analyzed the expression of ORs in 66 breast carcinoma tissues and cell lines and seven healthy breast tissues and ranked them according to their expression values (Figure 1). One receptor, OR2B6, had a pronounced expression in over 80% of all breast carcinoma tissues and 70% of all breast carcinoma cell lines, whereas it is not expressed in normal breast tissues (Figure 1C). OR2B6 was considered as “expressed” when FPKM value was >0.1. Expression values of OR2B6 ranged from FPKM 0.1 to 10.6, whereas an FPKM of 10.6 is remarkably high for an OR in non-olfactory tissues. OR2A7 and OR2W3 showed the second and third highest expression in the ranking, but there is, in contrast to OR2B6, a moderate expression in the healthy tissues as well. Beside OR2B6, OR56A3, OR4K15, OR2T8, OR4N4, OR8G5, OR2B2, OR13H1, OR7D2, and OR52N4 showed no expression in normal breast tissues, but were not uniformly expressed or even not expressed at all in most breast carcinoma tissues and cell lines.

### Expression of OR2B6 in Human Breast Carcinoma Tissues and Cell Lines

We detected OR2B6 in human breast carcinoma cell lines and in breast carcinoma tissues, but not in healthy tissues (Figure 1).

**Figure 2:**
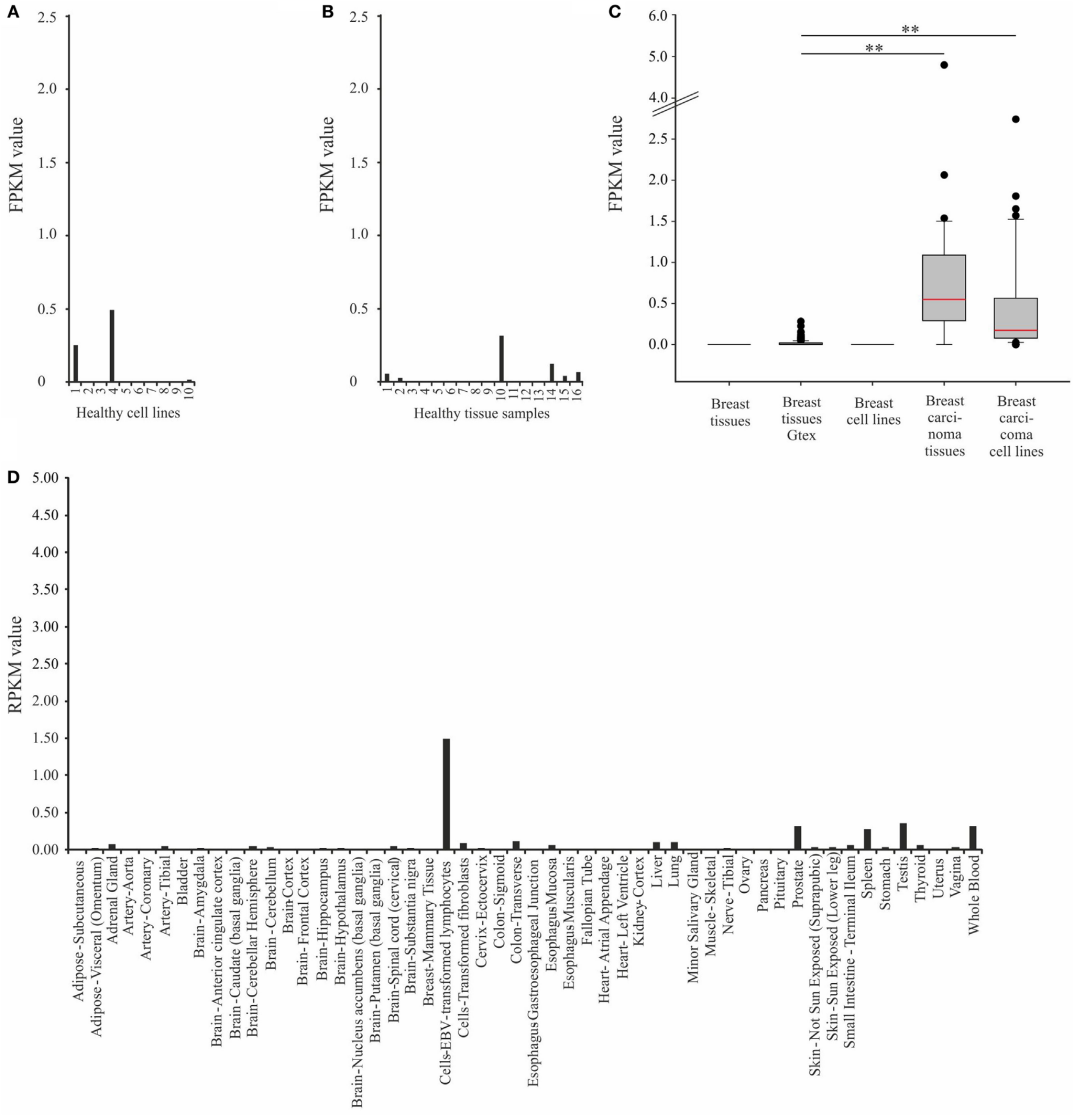
Expression analysis of OR2B6 in healthy tissue samples and cell lines. **(A)** Expression of OR2B6 in ten healthy cell lines originated from different human tissues. For further details see Table S2 in Supplementary Material. **(B)** Expression of OR2B6 in 16 different human tissues, origin: BodyMap Project (26). **(C)** Box-plot showing the expression of OR2B6 in normal breast tissues and breast carcinoma tissues and cell lines. Breast tissue: normal breast tissue samples, *n* = 7; breast tissue GTEx: normal breast tissue samples originated from GTEx database, *n* = 214; breast cell lines: cell lines originated from normal breast tissues, *n* = 4; breast carcinoma tissues: carcinoma breast tissue samples, *n* = 21; and breast carcinoma cell lines: cell lines originated from breast cancer tissues, *n* = 45. Normality test (Shapiro–Wilk) failed, Kruskal–Wallis one-way ANOVA: *p* < 0.05; the statistical significance is indicated as follows: **p < 0.01. Red line represents the median value. **(D)** Expression of OR2B6 in 52 healthy tissues originated from the GTEx database.

To quantify the expression of OR2B6, we analyzed the expression of OR2B6 in a larger amount of healthy breast tissue data (*n* = 214, origin: GTEx database) (Figure 2C). We confirmed the expression of OR2B6 in breast carcinoma tissues, whereas it was hardly detectable in healthy breast tissues (Figure 2C). Then, we analyzed the expression of OR2B6 in other healthy human cell lines (Figure 2A) and healthy tissue samples that originated from the BodyMapProject (Figure 2B) (26). Here, OR2B6 showed transcript expression with FPKM value <0.5 in 4 of 16 tissue samples. In addition, we investigated the expression in 52 different healthy tissues, originating from the GTEx database (Figure 2D). OR2B6 was only detectable in Epstein–Barr virus-transfected lymphocytes (median RPKM, 1.5), prostate (median RPKM, 0.3), spleen (median RPKM, 0.3), testis (median RPKM, 0.4), and whole blood (median RPKM, 0.3). Notably, 111 ORs are detectable in testis and several in Epstein–Barr virus-transfected lymphocytes (26).

**Figure 3:**
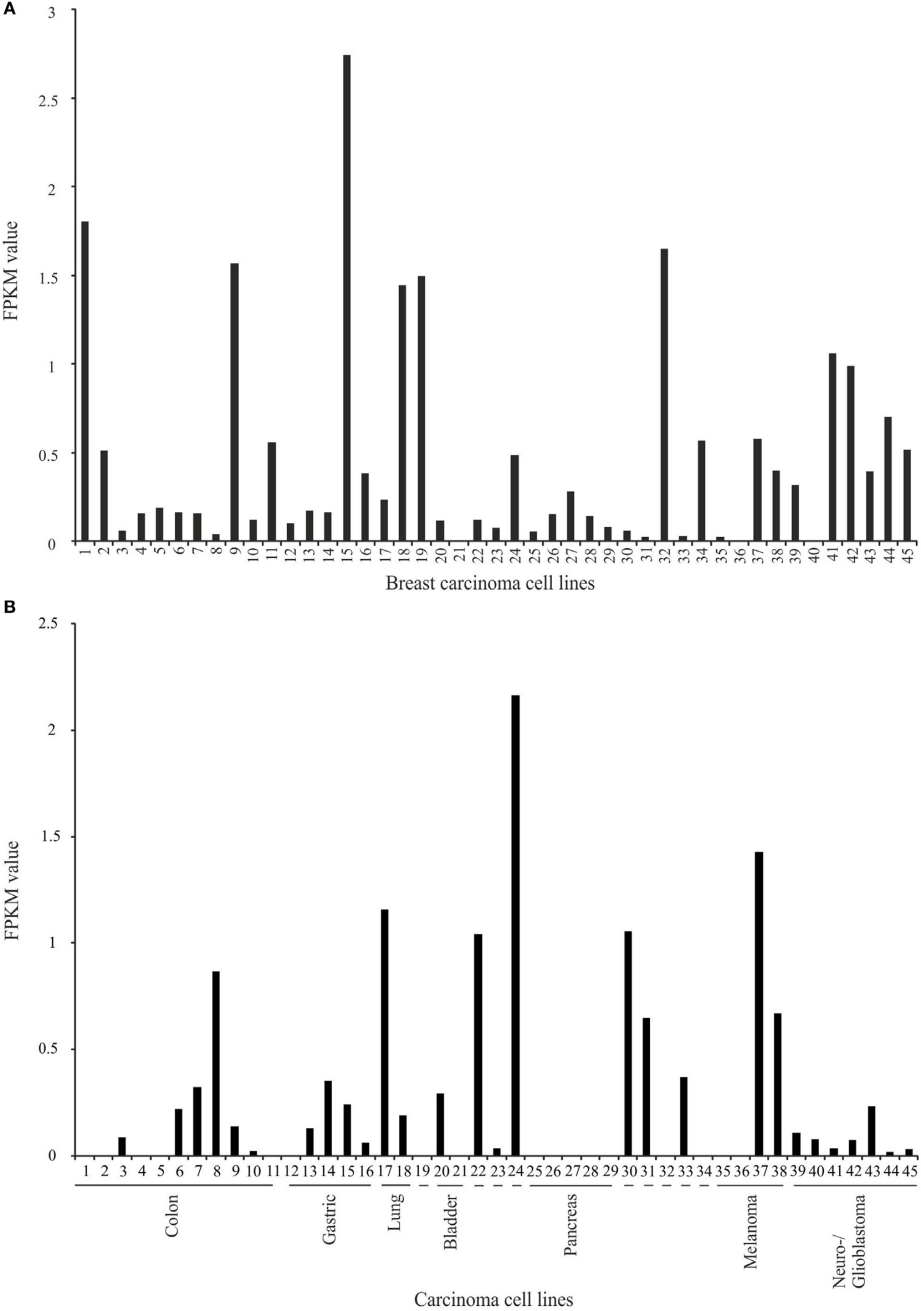
Expression analysis of OR2B6 in breast carcinoma cell lines and carcinoma cell lines that originated from other carcinoma types. **(A)** Expression of OR2B6 in 45 breast carcinoma cell lines. For further details on cell lines, see Table S1 in Supplementary Material. **(B)** Expression of OR2B6 in 45 other carcinoma cell lines, except breast carcinoma cell lines. 19: cell line that originated from osteosarcoma; 22: cell line that originated from prostate carcinoma; 23: cell line that originated from leukemia cells; 24: cell line that originated from germ cell carcinoma; 30: cell line that originated from cervix carcinoma; 31: cell line that originated from synovial sarcoma; 32: cell line that originated from Ewing sarcoma; 33: cell line that originated from myosarcoma; 34: cell line that originated from fibrosarcoma. For further details on cell lines, see Table S3 in Supplementary Material.

Thereafter, we focused on the expression of OR2B6 in breast carcinoma cell lines and cell lines that originated from other types of carcinoma (Figure 3). Here, 43 of 45 breast carcinoma cell lines do express OR2B6 transcripts (Figure 3A). OR2B6 is detectable in cell lines originated from other carcinoma types as well (Figure 3B), such as colon and gastric carcinoma cell lines, with FPKM values of up to 0.86, in a pluripotent embryonal carcinoma cell line (FPKM value: 2.16) as well as in a melanoma cell line (FPKM value: 1.42). In lung carcinoma tissue samples, there is a weak to moderate expression in two samples.

**Figure 4:**
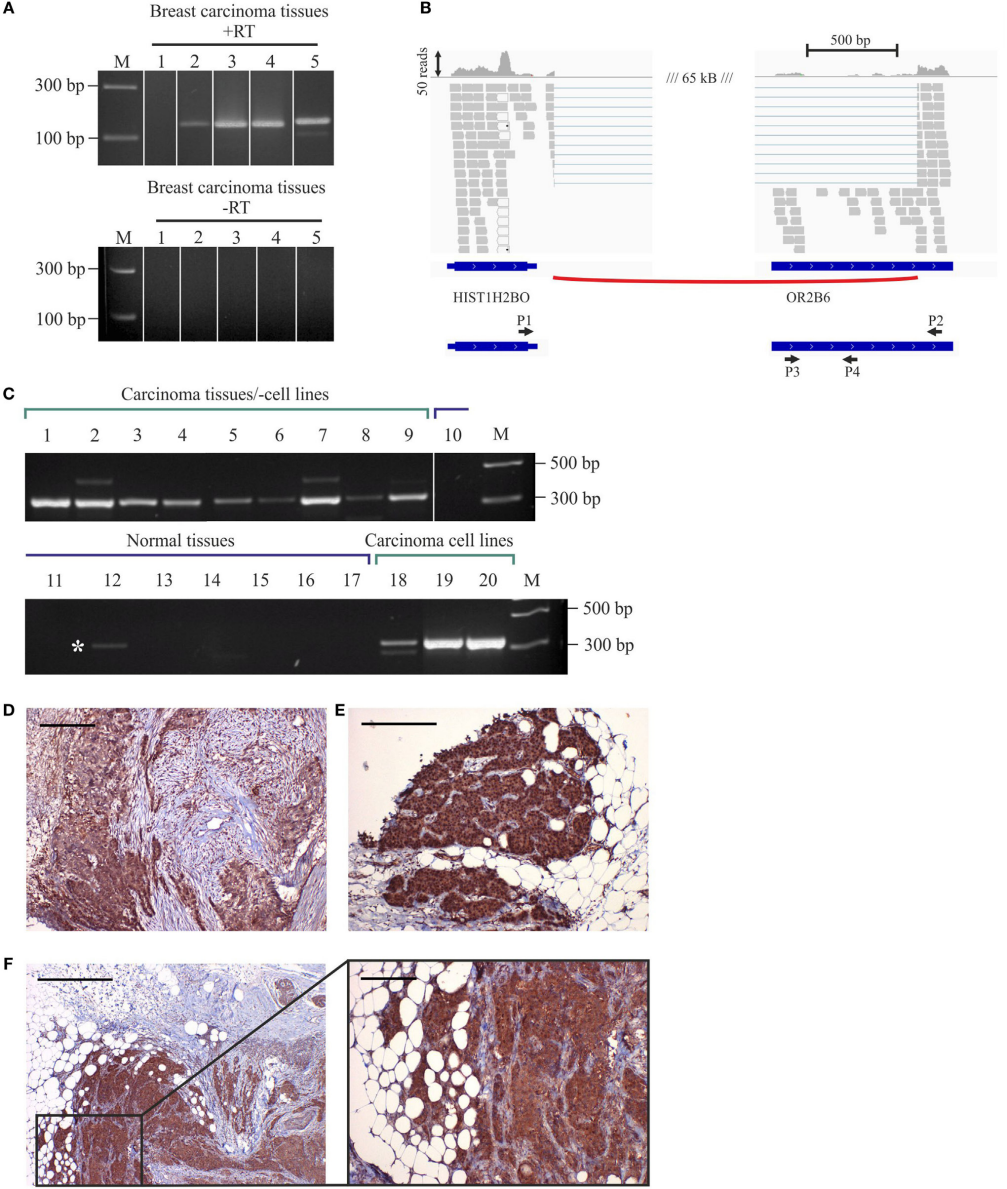
Expression of OR2B6 in human breast carcinoma tissues and detection of fusion transcripts by reverse transcriptase PCR (RT-PCR). **(A)** Validation of OR2B6 expression in five different breast carcinoma tissues *via* RT-PCR. +, +RT, cDNA; −, −RT, RNA; M, marker; bp, base pairs. **(B)** Visualization of the fusion transcript of OR2B6 and HIST1H2BO *via* the integrative genomic viewer. **(C)** Validation of the expression of the fusion transcript of OR2B6 and HIST1H2BO *via* RT-PCR. +, +RT, cDNA; −, −RT, RNA; M, marker; bp, base pairs. **(D)** Immunhistochemical staining of metaplastic breast carcinoma tissue using specific human α-OR2B6 antibody. Tumor details: G3, triple negative. Shown is the expression of the receptor protein in a triple-negative metaplastic breast carcinoma in G3. Cancer cells are specifically stained, surrounding connective tissue fibers and fat cells show no staining. **(E)** Immunhistochemical staining of invasive ductal breast carcinoma tissue using specific human α-OR2B6 antibody. Shown is the expression of the receptor in an E^+^/PR^−^/HER2^−^ carcinoma in G2 (estrogen 100%: ER^+^, progesterone 0%: PR^−^, Her2 negative: HER2^−^). Protein expression is visualized using DAB chromogenic staining and co-staining with hematoxylin (HE) is used to illustrate the tissue architecture. **(F)** Immunhistochemical staining of invasive ductal breast carcinoma tissue using specific human α-OR2B6 antibody. Tumor details: G3, triple negative. Shown is the expression of the receptor protein in a triple-negative invasive ductal breast carcinoma in G3. Scale bar: 100 µm.

To validate the NGS sequencing data of OR2B6 transcript expression, we confirmed the expression of OR2B6 in native breast carcinoma tissues via RT-PCR (Figure 4A) and observed the distribution of mapped reads using IGV. Thereby, we detected an interesting read distribution. Both the coding sequence of OR2B6 and a histone transcript, HIST1H2BO, were covered with reads and are connected by a splice junction (Figure 4B). HIST1H2BO is a member of the histone cluster 1 H2B family. Family member E of the same cluster was found to be relevant in endocrine-resistant breast cancer (45). ORs are capable of building fusion transcripts with transcripts from upstream genes (26). We carried out an RT-PCR with exon spanning primers between OR2B6 and HIST1H2BO and could verify the expression of this fusion transcript (Figure 4C). Interestingly, this fusion transcript was detected in all breast carcinoma tissue samples *via* RT-PCR, whereas we could not detect it in healthy tissues although there was one exception. Our analyses show the expression of two transcripts. First, the expression of a transcript coding for OR2B6, which was supported by reads covering the complete coding sequence and the detection of the transcript with PCR primers P3 and P4. Second, the expression of a HIST1H2BO splice variant, which contains parts of OR2B6 as a downstream exon, detected by primers P1 and P2 and by exon spanning reads in the NGS sequencing. We analyzed the OR2B6 protein in native breast carcinoma tissue samples by performing immunohistochemical staining of breast carcinoma tissue samples using a specific antibody detecting OR2B6. Specificity of OR2B6 was confirmed by immunocytochemistry of OR2B6-transfected Hana3A cells using antibodies detecting OR2B6 and a rhodopsin tag located in the OR2B6-containing plasmid (Supplement Figure S1 in Supplementary Material). Consistently, OR2B6 expression was detectable in breast carcinoma cells (Figures 4D–F).

Thus, our data indicate that OR2B6 expression is associated with breast carcinoma and therefore might be considered as a potential tumor marker. Further analyses need to elucidate whether OR2B6 has a functional role in breast carcinoma as well.

### Expression of OR2B6 and Other ORs in Different Human Carcinoma Tissues

**Figure 5:**
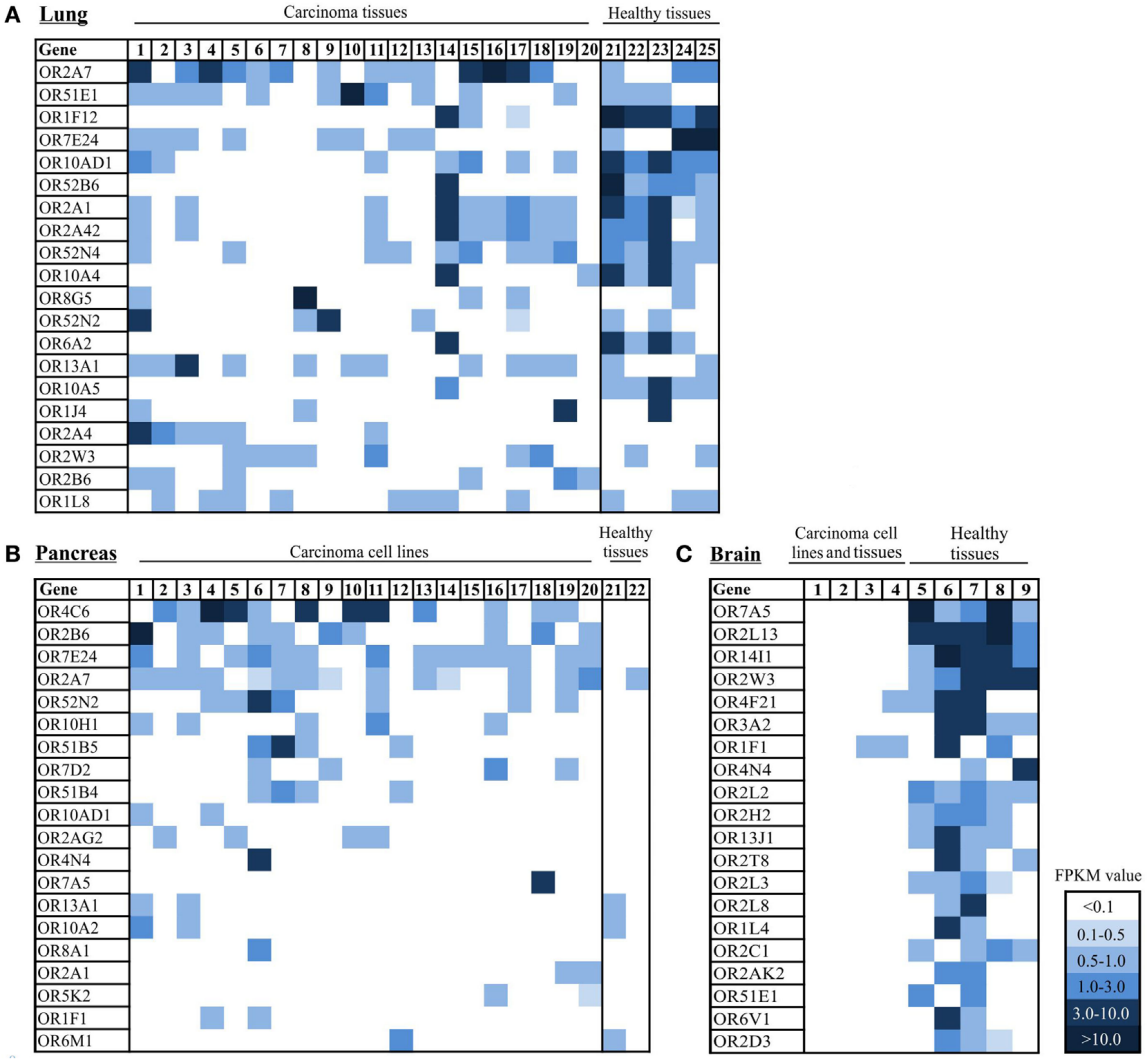
Expression pattern of ectopically expressed olfactory receptors (ORs) in lung, pancreas, and brain carcinoma tissue samples, cell lines, and healthy tissues. **(A)** Heat map shows 20 ranked ORs according to their expression level in 20 lung carcinoma tissues in comparison to 5 healthy lung tissues. 1–20: lung carcinoma tissue samples; 21–25: normal lung tissue samples. **(B)** Heat map shows 20 ORs ranked according to their expression level in 20 pancreatic cancer tissues and cell lines in comparison to 2 healthy pancreas tissues. 1: Cell line *Capan-1*. 2–20: patient-derived pancreatic carcinoma cell lines. 21–22: healthy pancreas tissue samples. **(C)** Heat map shows 20 ORs ranked according to their expression level in 4 brain tumor tissue samples and cell lines and 5 healthy brain tissue samples. 1–3 brain tumor cell lines, 4: brain carcinoma tissue sample, 5–9: healthy brain tissue samples. Color intensity correlates with the expression strength. Dark blue represents FPKM values >3, and light blue represents FPKM values <0.5. For further details on cell lines, see Table S4 in Supplementary Material.

While detecting OR2B6 transcripts in several cell lines that originated from different carcinoma tissues (Figure 3), we investigated the expression profile of OR2B6 in lung, pancreas, and brain carcinoma tissue samples. For the analysis of the OR expression profile in lung carcinoma, 5 normal lung tissues and 20 lung carcinoma tissues were investigated. In total, 20 ORs were ranked due their expression level (Figure 5A). Strikingly, the ORs expressed in the healthy lung in general show a higher expression level than the ORs in lung carcinoma tissues. OR2A4/7 shows, on average, the highest expression in most of the cancerous samples, with FPKM values ranging from 0.2 to 5.0. OR51E1 is detectable in both normal and cancerous tissues, whereas some ORs, such as OR1F12 and OR10AD1, are predominantly expressed in healthy lung tissues. Interestingly, OR6A2 and OR10A5 are only expressed in healthy tissues, with one exception.

Analysis of the expression pattern of ORs in 20 pancreas carcinoma tissues and cell lines revealed increased expression of OR4C6 (Figure 5B). This expression was detectable in 12 of 20 samples, with an FPKM value of 0.1–6.2. The verification of OR4C6 expression *via* the IGV is shown in Figure S2 in Supplementary Material. By analyzing the expression of OR4C6 in 52 healthy human tissues from the GTEx portal, it was striking that OR4C6 showed a median expression of 0.0 RPKM in all tissue samples (data not shown), which indicates that it may represent a possible biomarker for pancreatic carcinoma. The second highest expression was observed for OR2B6, which was expressed in 10 of 20 pancreatic carcinoma samples. Interestingly, the expression of both receptors was restricted to cancerous tissues. OR7E24 and OR2A7 were also expressed, but were not specific to carcinoma tissues in comparison to normal pancreas tissues.

Furthermore, we observed the expression of ORs in brain tissue and brain tumor tissue (Figure 5C). It was striking that there were only three ORs (OR4F21, OR1F1, and OR2B6) expressed in cancerous tissues and cell lines, whereas several ORs were expressed in three different healthy brain tissues (Figure 5C). OR14I1 and OR2L13 showed the highest expression, with FPKM values ranging from 1 to 10 (Figure 5C).

## Discussion

In this study, we first analyzed the expression profile of ORs in human breast carcinoma tissues. As far as we know, there is no comprehensive data concerning the expression pattern of ORs in human breast carcinoma tissues. Here, we used RNA-Seq analysis, which allows for the investigation of a large amount of data to reveal conclusive expression information. Our analyses of breast carcinoma tissue samples and cell lines revealed that OR2B6 is nearly solely expressed in cancerous tissues and therefore might represent a tumor marker. We demonstrated its expression in breast carcinoma tissues (represented in over 80% of the samples) and breast carcinoma cell lines (represented in 73% of the samples), but almost not in healthy breast tissue samples. The few breast carcinoma samples, which did not elicit OR2B6 expression, require further investigation. We did not observe different breast cancer subtypes, as the aim of our study is to demonstrate the overall expression of OR2B6 in breast cancer compared with healthy tissues. In further studies, we will investigate the expression of OR2B6 in different breast cancer subtypes. We analyzed data from a study that was conducted to detect biomarkers in blood platelet samples from patients suffering different cancer types; here, we found OR2B6 to be upregulated in tumor-educated blood platelets from patients suffering from breast cancer, so that OR2B6 might function as a biomarker that can be diagnosed *via* blood samples (46).

Immunohistochemical analyses confirmed the existence of OR2B6 at the protein level and revealed a distinct staining in native breast carcinoma tissues. Here, analyses of further tissue samples will elucidate whether OR2B6 functions as a tumor marker like OR51E1 and OR51E2 (14, 22, 47). It is important to mention that OR2B6 unlike OR51E1 and OR51E2 lacks expression in healthy tissues, ensuring that the high expression does not come from an increase of a specific cell type but from the existence of the different transcript repertoire in cancer cells. In addition, we showed that OR2B6 builds a fusion transcript with a histone gene, HIST1H2BO, in several carcinoma tissues. The building of fusion transcripts was already shown for OR2W3, OR4N4, and OR2T8, which build chimeric transcripts with the Trim58 gene and part of loc728377 (26). Volz et al. demonstrated six different transcriptional start sites combined with alternative splicing for one OR gene, exhibiting a region of 81 bp with potential bidirectional transcriptional activity (48). Although the function of such ORs building fusion transcripts is far from understood, studies have already postulated that OR genes that are expressed in diverse tissues need different promoters to ensure flexible transcriptional regulation (48). Chimeric transcripts found *via* RNA-Seq analyses could be successfully detected on the protein level (49). By using validation experiments, we are sure that both the transcript of the OR2B6 gene and the fusion transcript are expressed. The latter could be of great importance, as histones are known to be the most conserved human proteins (50). Apart from that, a mutation in the HIST1H1A complex (which comprises HIST1H2BO) is associated with breast cancer and the expression of histone H2B is increased in 70% of breast cancer tissues in comparison to the expression in normal breast tissues (51, 52). The expression of OR2B6 correlates with the expression of the HIST1H2BO-OR2B6-fusion transcript, which thereby might influence the functionality of HIST1H2BO. The function of this interesting fusion transcript will be part of future studies. Normally, replication-dependent histone mRNAs are not polyadenylated (53). However, this splicing event will probably result in a polyadenylated histone transcript as it was shown for HIST1H2BD and HIST1H2AC (53). The occurrence of such polyadenylated histone transcripts is influenced by the differentiation status of a variety of cell types and is also increased following the induction of double-strand DNA breaks *via* gamma-irradiation (54).

Unfortunately, the majority of ORs is orphan, as the screening for ligands is highly complex and plethora of odorants exists. Not least because of the high number of potential odorants but also because of the obstacles of transfecting ORs in heterologous cell systems to screen for possible ligands, deorphanization of ORs is highly complicated. Due to the lack of an activating ligand for OR2B6, we cannot investigate the effect of the receptors functionality, which would be of great interest.

Furthermore, we investigated the expression of OR2B6 in other carcinoma tissues and corresponding healthy tissues. OR2B6 was not detectable in healthy tissue samples, with only a few exceptions that had low expression values. We could detect OR2B6 in the lung and in pancreas carcinoma tissues as well, whereas in the latter, OR2B6 shows the second highest expression. The highest expression was demonstrated for OR4C6, which had a moderate expression in several pancreatic carcinoma tissues, but not in healthy pancreas tissue. Interestingly, the median expression of OR4C6 in all healthy tissue samples listed in the GTEx database was RPKM 0. The importance of ORs for pancreatic cancer was shown by a genome-wide association study, which associated the olfactory transduction pathway with pancreatic cancer risk. OR13C4 and OR13C3 were the third and fifth most significant genes among the 5,127 genes analyzed (55). Sequencing analysis of human pancreatic cancer revealed several somatic mutations in OR genes, such as OR13C3, OR13C5, OR10P1, OR1J2, OR4A16, OR51F2, and OR5D13 (56). We did not investigate single mutations, but the overall expression level of the ORs investigated by Wei et al. and did not detect a considerable difference (55). Strikingly, OR4C6 was shown to be influenced by parts of the supraspliceosome, which is the Ran-binding domain containing protein 2 (ZRANB2) that acts on the alternative splicing of OR4C6 (57). As ZRANB2 is upregulated in types of ovarian carcinoma and acts on the alternative splicing of genes implicated in tumor development, we suggest a role for OR4C6 in the development of pancreatic cancer (57). Likewise, the functional role of OR2C3 in the development or progression of melanomas was shown by Ranzani et al. (25).

In lung carcinoma tissues, OR2B6 was expressed in 30% of all samples, excluding OR2B6 as a reliable tumor marker for this carcinoma type. Nevertheless, it is remarkable that OR2B6 was not detectable in any healthy lung tissue samples.

In the remaining carcinoma tissue samples and cell lines that originated from brain carcinomas, OR2B6 was not detectable.

Our findings indicate that ORs could play an important role as potential biomarkers in the field of cancer research, and a growing body of data also supports this hypothesis. OR51E2 is known to function as a biomarker for prostate cancer, and its paralog, OR51E1, was discovered as a potent biomarker for the small intestine and some types of lung cancer (13, 14, 22, 23). Another biomarker for colon cancer is OR7C1; its expression functions as a prognostic marker for colorectal cancer (24). Recently, a study by Kalbe et al. revealed the importance of ORs in non-small-cell lung cancer and its importance for innovative cancer therapy (16). To improve the sensitivity of OR2B6 for breast carcinoma, we will correlate OR2B6 expression with clinical features like tumor grading, stage, and Heregulin2 status in upcoming studies.

In summary, we identified a potential biomarker for breast carcinoma tissues, OR2B6. Of course, we cannot assure the suitable specificity of OR2B6, but based on our previous study, we are highly confident that this receptor might represent a powerful tool for differentiating between healthy and cancerous breast tissues or even other tissues, as OR2B6 can be detected in pancreas and lung carcinoma tissues as well but not in the corresponding healthy tissues.

## Ethics Statement

This study was carried out in accordance with the recommendations of the Regional Research Ethics Committee (Ethical Committee of the Medical Faculty, Ruhr-University Bochum), with written informed consent from all subjects. All subjects gave written informed consent in accordance with the Declaration of Helsinki. The protocol was approved by the Ethical Committee of the Medical Faculty, Ethics No. 17-6113.

## Author Contributions

Conceptualization: LW, HH, GG, and DM. Formal analysis: LW, GG, HH, CB, JA, and DM. Investigation: LW, CB, JA, BU, GB, GW, SP, and AT. Writing (original draft preparation): LW and DM. Writing (review and editing): GG, CB, JA, BU, GB, GW, SP, and AT. Visualization: LW, DM, SP, and AT. Supervision: LW, GG, AT, and SP. Project administration: HH.

## Conflict of Interest Statement

The authors declare that the research was conducted in the absence of any commercial or financial relationships that could be construed as a potential conflict of interest.
