# Supplementary material for: Olfactory Receptors as Biomarkers in Human Breast Carcinoma Tissues
Source: Front Oncol. 2018 Feb 15;8:33. doi: 10.3389/fonc.2018.00033 (PMC5818398; doi:10.3389/fonc.2018.00033)
Supplement: Supplementary file 1 [file data_sheet_1.docx]

Supplement

Supplement Figure 1:

**Supplement Figure 1: Immuncytochemical staining of Hana3A cells transfected with OR2B6 using a specific α-OR2B6-antibody (red) and an antibody detecting the α-rho-tag (green).** For nuclear staining of the cells, 4´6-diamidino-2-phenylindole (DAPI; dilution 1:200) was applied. Scale bar: 10 µm.

**Supplement Figure 2: Visualization of the expression of OR4C6 via the Integrative Genomic Viewer.** OR4C6 gene is indicated by the blue box. The grey segments represent reads that are mapped onto the reference genome hg19. The read coverage is shown above the mapped reads.

Supplement Table 1:

List of breast carcinoma cell lines:

| 1 | T47D |
| --- | --- |
| 2 | BT474 |
| 3 | MCF-7 |
| 4 | HCC1954 |
| 5 | SUM225 |
| 6 | SUM131502 |
| 7 | HCC3153 |
| 8 | SUM102 |
| 9 | HCC1937 |
| 10 | MDAMB453 |
| 11 | SUM149PT |
| 12 | MDAMB231 |
| 13 | HCC2157 |
| 14 | SUM185 |
| 15 | ZR7530 |
| 16 | SW527 |
| 17 | SUM190 |
| 18 | UACC893 |
| 19 | UACC3199 |
| 20 | SKBR5 |
| 21 | OCUBM |
| 22 | MFM223 |
| 23 | SKBR7 |
| 24 | UACC812 |
| 25 | MDA361 |
| 26 | MDAMB415 |
| 27 | MB157 |
| 28 | KPL15 |
| 29 | MDAMB330 |
| 30 | JIMT1 |
| 31 | HCC1143 |
| 32 | HCC518 |
| 33 | HBL51 |
| 34 | CAM1 |
| 35 | EFM19 |
| 36 | EVSAT |
| 37 | DU4475 |
| 38 | CAL148 |
| 39 | BT549 |
| 40 | CAL120 |
| 41 | CAL51 |
| 42 | MX1 |
| 43 | SUM44 |
| 44 | SUM52N |
| 45 | HTBG9 |

Supplement Table 2:

List of healthy human cell lines:

| 1 | MAQ cells |
| --- | --- |
| 2 | HK2 |
| 3 | Huvec |
| 4 | PrEC |
| 5 | BEA2B |
| 6 | BJFibro |
| 7 | HME |
| 8 | HMEC |
| 9 | MCF10A |
| 10 | IMR90 |

Supplement Table 3:

List of other cancerous cell lines:

| 1 | HCT116 |
| --- | --- |
| 2 | CACO-2 |
| 3 | DLD-1 |
| 4 | HCT-15 |
| 5 | RKO |
| 6 | SW48 |
| 7 | HT29 |
| 8 | LS1034 |
| 9 | SW480 |
| 10 | COLO 205 |
| 11 | LS174T |
| 12 | YCCEL1 |
| 13 | SNU719 |
| 14 | SNU638 |
| 15 | NUGC-3 |
| 16 | NCC-24 |
| 17 | NCI-H1299 |
| 18 | A549 |
| 19 | SAOS-2 |
| 20 | 5637 |
| 21 | T24 |
| 22 | LNCaP |
| 23 | THP-1 |
| 24 | NCCIT |
| 25 | YAPC |
| 26 | MIAPaCa-2 |
| 27 | PT45 |
| 28 | PA-TU8988 |
| 29 | PANC-1 |
| 30 | HeLa |
| 31 | SW982 |
| 32 | TTC-466 |
| 33 | SCMC-RM2-1 |
| 34 | HT-1080 |
| 35 | SK-ML-5 |
| 36 | HCT21023 |
| 37 | BLM |
| 38 | WM1366 |
| 39 | U-251 |
| 40 | U87 |
| 41 | IMR-32 |
| 42 | U-343 |
| 43 | SK-N-SH |
| 44 | LN-229 |
| 45 | SH-EP |

Supplement Table 4:

List of brain carcinoma cell lines:

| 1 | U343 |
| --- | --- |
| 2 | LN229 |
| 3 | GBM1B |
| 4 | U87 |
