# Supplementary figures and images for: Olfactory Receptors as Biomarkers in Human Breast Carcinoma Tissues
Source: Front Oncol. 2018 Feb 15;8:33. doi: 10.3389/fonc.2018.00033 (PMC5818398; doi:10.3389/fonc.2018.00033)

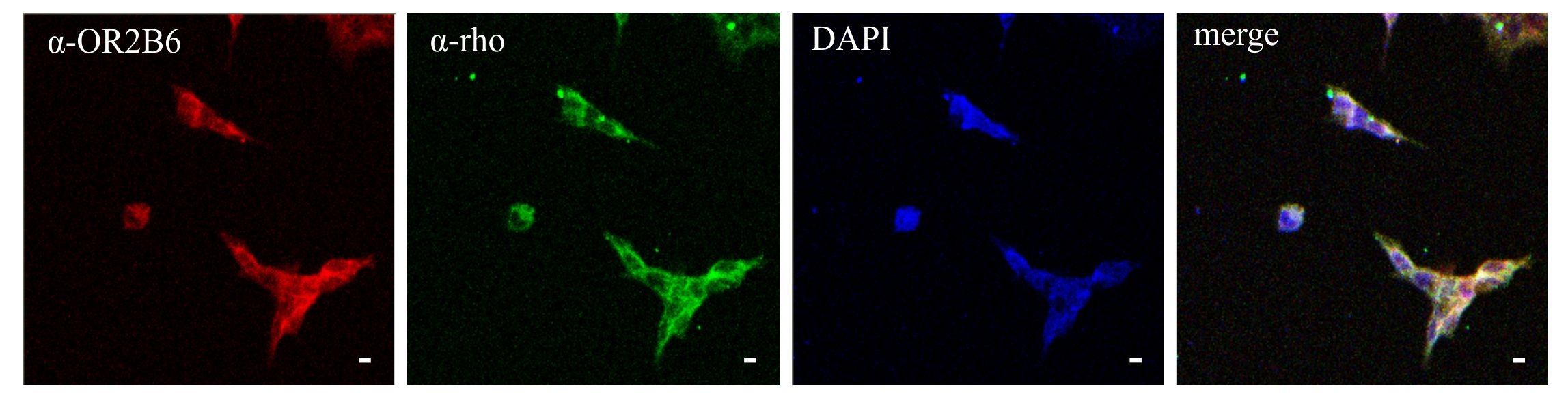

Supplement: Supplementary file 2 [file image_1.jpeg]

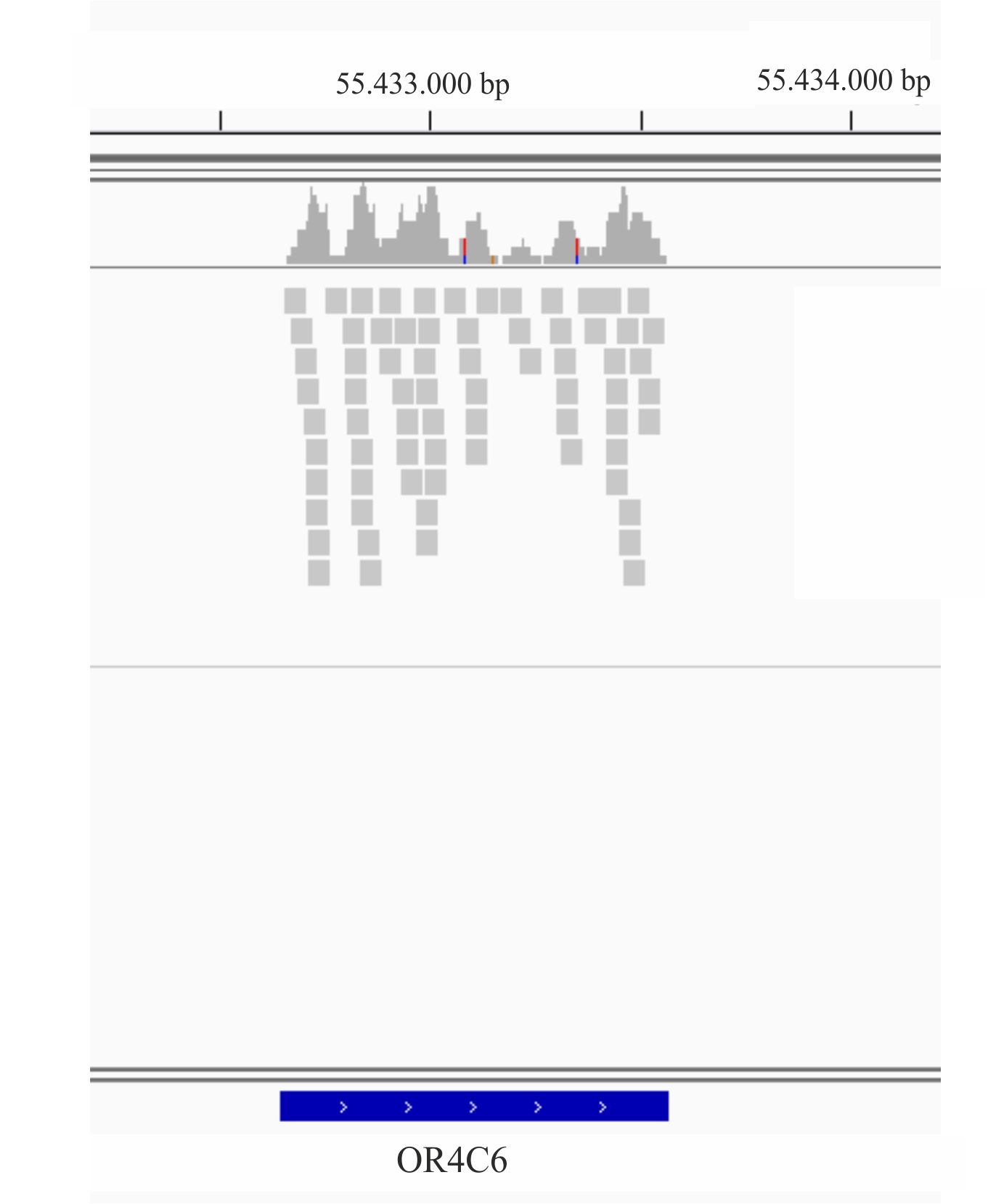

Supplement: Supplementary file 3 [file image_2.jpeg]
